# Does Proprioception Influence Human Spatial Cognition? A Study on Individuals With Massive Deafferentation

**DOI:** 10.3389/fpsyg.2018.01322

**Published:** 2018-08-07

**Authors:** Alix G. Renault, Malika Auvray, Gaetan Parseihian, R. Chris Miall, Jonathan Cole, Fabrice R. Sarlegna

**Affiliations:** ^1^Aix Marseille Univ, CNRS, ISM, Marseille, France; ^2^Sorbonne Université, UPMC, CNRS, Institut des Systémes Intelligents et de Robotique (ISIR), Paris, France; ^3^Aix Marseille Univ, CNRS, PRISM, Marseille, France; ^4^School of Psychology, University of Birmingham, Birmingham, United Kingdom; ^5^Clinical Neurophysiology, Poole Hospital, and Centre of Postgraduate Research and Education, University of Bournemouth, Poole, United Kingdom

**Keywords:** space representation, mental model, reference frame, proprioception, sensory neuropathy

## Abstract

When navigating in a spatial environment or when hearing its description, we can develop a mental model which may be represented in the central nervous system in different coordinate systems such as an egocentric or allocentric reference frame. The way in which sensory experience influences the preferred reference frame has been studied with a particular interest for the role of vision. The present study investigated the influence of proprioception on human spatial cognition. To do so, we compared the abilities to form spatial models of two rare participants chronically deprived of proprioception (GL and IW) and healthy control participants. Participants listened to verbal descriptions of a spatial environment, and their ability to form and use a mental model was assessed with a distance-comparison task and a free-recall task. Given that the loss of proprioception has been suggested to specifically impair the egocentric reference frame, the deafferented individuals were expected to perform worse than controls when the spatial environment was described in an egocentric reference frame. Results revealed that in both tasks, one deafferented individual (GL) made more errors than controls while the other (IW) made less errors. On average, both GL and IW were slower to respond than controls, and reaction time was more variable for IW. Additionally, we found that GL but not IW was impaired compared to controls in visuo-spatial imagery, which was assessed with the Minnesota Paper Form Board Test. Overall, the main finding of this study is that proprioception can influence the time necessary to use spatial representations while other factors such as visuo-spatial abilities can influence the capacity to form accurate spatial representations.

## Introduction

Humans can form mental models of spatial environments based on a variety of materials such as written or verbal information (Tversky, 1991; Bryant et al., 1992; Denis and Zimmer, 1992; Taylor and Tversky, 1992; Noordzij and Postma, 2005). A mental model can represent temporal or causal features of an event and may be used to infer some spatial cues, even when the initial description does not explicitly describe spatial relations (Denis and Zimmer, 1992; Rinck et al., 1996; Bestgen and Dupont, 2003). Spatial mental models may include landmarks, geometric properties of the represented environment, and distances (Noordzij and Postma, 2005).

The reference frame (or perspective) used in a description appears to influence the formation of a spatial model (Taylor and Tversky, 1992; Noordzij and Postma, 2005, see also Gallay et al., 2013, for a review). The main reference frames which have been considered in the literature are the egocentric reference frame (sometimes referred to as route perspective) and the allocentric reference frame (sometimes referred to as survey perspective). The egocentric reference frame is based on the point of view of the addressee, that is, in first person. On the other hand, for the allocentric reference frame, knowledge of the environment is built from a bird-eye’s point of view, independent of the addressee’s viewpoint. Thus, each object’s position can be represented relative to the position of another object using cardinal information. In Tversky (1991) pioneering work, the influence of the reference frame on spatial representations was assessed by manipulating the spatial description. Tversky (1991) termed the route (or egocentric) reference frame condition when objects were described to an observer as a function of his/her position, in a sequential way, and using the second-person singular form, with terms such as “to your left,” “to your right,” “in front of you,” and “behind you.” In the survey (or allocentric) reference frame condition, objects were presented in the description with terms such as “to the north” or “to the east.” Tversky (1991) and Taylor and Tversky (1992) reported that spatial models were similar whether the environment was described according to an allocentric or to an egocentric reference frame. However, when Noordzij and Postma (2005) studied the ability of healthy individuals to infer the distance between objects after listening to verbal descriptions, they found a relative advantage for the allocentric reference frame (survey-type description) compared to the egocentric reference frame (route-type description). Therefore, while it is well established that metric distance information can be derived from verbal descriptions formatted according to either an egocentric or an allocentric reference frame, the link between reference frames and spatial models remains unclear.

The way in which sensory experience influences spatial cognition has been investigated and it is now well established that visual information influences spatial models (see Arnold et al., 2017a, for a review). For instance, many studies have compared the performance of sighted and blind individuals in spatial tasks. After Leonard and Newman (1967) first reported that blind individuals can form and use mental models for spatial navigation, Rieser et al. (1980) reported that blind people can perform as well as sighted people in spatial cognition tasks. It has been hypothesized that blind people compensate for their lack of vision by means of other sensory modalities and with substantial brain reorganization, for instance in the visual cortex whose plasticity has been highlighted (Sadato et al., 1996; Cohen et al., 1997). Both in a listening task (Kujala et al., 2000) and a voice perception task (Gougoux et al., 2009), the visual cortex was found to be activated in blind participants, in contrast to control participants, thus appearing to contribute to the processing of auditory inputs. Brain plasticity may explain how blind people can outperform sighted people in auditory perception and tactile discrimination (Stevens et al., 1996; Lessard et al., 1998; Van Boven et al., 2000; Wong et al., 2011). This suggests that after a massive sensory loss, use-dependent plasticity can lead to substantial reorganization in the central nervous system (Pons et al., 1991) and even to functional advantages in tasks involving other sensory modalities (Cole et al., 1995; ter Horst et al., 2012; see also Bavelier et al., 2006).

Noordzij et al. (2006) studied the influence of the reference frame on blind people’s ability to form and use spatial models. After listening to a verbal description formatted according to an allocentric reference frame, blind people performed worse than sighted people in a distance-comparison task. However, they performed better than sighted people when an egocentric reference frame was used. Ruggiero et al. (2009) also showed that for a task involving allocentric and egocentric judgments, congenitally blind people performed worse than sighted participants in allocentric judgments. In line with that study, Pasqualotto et al. (2013) investigated the preferred reference frame of spatial memory for congenitally blind, late blind, and sighted people. The congenitally blind people preferentially used an egocentric reference frame, while late blind and blindfolded sighted people preferentially used an allocentric reference frame. Thus, reference frame seems dependent on the – current or past – access to visual information (see Arnold et al., 2017b, for a review).

While the influence of vision on human spatial cognition has been widely investigated, little is known about the influence of proprioception. Proprioception is the sense of position and movement of body segments, based on signals from muscles, tendons, joints, and skin (Cole, 2016). Rare individuals who have permanently lost proprioception (due to a sensory neuronopathy; Cole and Paillard, 1995) have been studied but very little has been done to assess the influence of proprioceptive loss on cognitive skills. Most of the research conducted so far on deafferented individuals has focused on their motor impairments and showed that deafferented individuals can compensate, at least partly, for their motor deficit by using vision and attention (Blouin et al., 1993; Sainburg et al., 1993; Cole et al., 1995; Ghez et al., 1995; Ingram et al., 2000; Sarlegna et al., 2010). Blouin et al. (1993) reported that reaching arm movements of a deafferented individual (identified as GL) were as accurate as those of control individuals when vision was available, while her errors were greater than controls when vision was removed. Blouin et al. (1993) suggested that the loss of proprioception results in an impaired egocentric frame of reference, and that, in healthy individuals, the egocentric reference frame is continuously updated based on static and dynamic proprioceptive signals, as previously suggested by Paillard (1987).

Since the loss of proprioception has been suggested to impair the egocentric frame of reference, at least in a study of reaching movements (Blouin et al., 1993), we hypothesized that deafferented individuals would be impaired in their ability to form or use spatial representation compared to controls when the spatial environment is described in an egocentric reference frame. Such impairment may be characterized by an increase in the mean value, or the variability, of the error score and/or the reaction time. In contrast, based on a study which suggested that a deafferented participant (GL) may exclusively rely on an allocentric reference frame in a perceptual, Rod-and-Frame Test (Bringoux et al., 2016), and based on Noordzij et al. (2006) findings, we also hypothesized that deafferented individuals might perform better than controls when the spatial environment is described in an allocentric reference frame. At last, since proprioceptive loss has been shown to impact the timing in sensorimotor tasks (Bard et al., 1992; Sainburg et al., 1993) as well as the reaction time to auditory stimuli while walking (Lajoie et al., 1996), we hypothesized that reaction time may be increased, or more variable, for deafferented individuals compared to controls.

## Materials and Methods

### Participants

Two deafferented participants, massively deprived of proprioception (GL, a 67-year-old woman; IW, a 62-year-old man) and 16 control participants (mean age of 62 years, ranging between 45 and 73, nine men and seven women) completed the experiment. This study was carried out in accordance with the recommendations of the institutional review board of the Institute of Movement Sciences. The protocol was approved by the institutional review board of the Institute of Movement Sciences. All the participants gave written informed consent in accordance with the Declaration of Helsinki. All the participants were naive to the purpose of the experiment.

A case-group comparison (Michael, 2007) showed no significant difference in age between deafferented participants and the control participants (*Q*′ = 1.54; *p* > 0.05 for GL and *Q*′ = −0.13; *p* > 0.05 for IW). None of the control participants reported having neurological, motor, or proprioceptive deficits. Case reports of GL and IW have been described in several articles (Cole and Paillard, 1995; Forget and Lamarre, 1995; Lefumat et al., 2016; Miall et al., 2018). To summarize their impairment, GL and IW suffered from an acute sensory neuronopathy when they were 31 and 19 years old, respectively: this resulted in the specific loss of large-diameter, Aα and Aβ myelinated afferents. Since then, they have lost all somatosensory modalities (kinesthesia, tendon reflexes, touch, vibration, and pressure). In particular, they have lost position and movement sense of all body parts, from nose down for GL and from neck down for IW. Small sensory fiber function, pain and temperature were not affected and neither were the motor nerves. Motor abilities seemed immediately incapacitated and both participants required years of training to develop some controlled movements. GL has used a wheelchair since. IW learned to stand and then walk again but, a few years ago, a persistent back problem, and the mental effort required for standing, led him to use a wheelchair as well.

### Task

Participants listened to a recorded description of a spatial map through a headset (Amarina GH1860) connected to a laptop (ASUS serial E570). The description was in participant’s primary language (English for IW, French for all the others). It should be mentioned that spatial processing appears to be similar whether descriptions are in French (Mellet et al., 2002) or English (Taylor and Tversky, 1992) since, for an identical description, the percentage of correct responses in a recall phase is similar (82–90 and 81–89%, respectively). In addition, the symbolic distance effect was found to be significant whether the description was in French (Denis, 2008) or in English (Moyer and Bayer, 1976). The experiment was run on an interface implemented in Matlab (Mathworks, Natick, MA, United States), allowing the pre-recorded vocal descriptions to be played and to record the participants’ responses.

**FIGURE 1:**
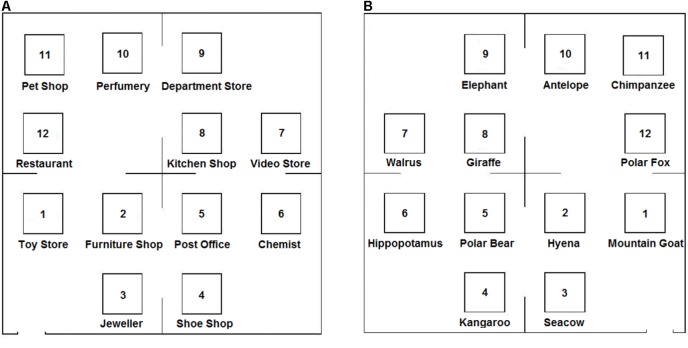
Fictitious map of the Shopping Center **(A)** and the Zoo **(B)**. Participants had to imagine the location of 12 objects based on an auditory description which was repeated six times. They could see at the end of the experiment the corresponding, blank map of the environment, with 12 empty boxes to perform a free-recall task.

As in Noordzij et al. (2006) study, the descriptions were fictitious grid-like maps of either a shopping center or of a zoo (**Figure 1**), with two types of descriptions: one according to an egocentric (route) reference frame and the other according to an allocentric (survey) reference frame (see **Appendix** for examples). The descriptions differed on several points. For instance, in the allocentric description; objects were introduced in relation to a previously mentioned object, whereas the egocentric description referred to participants in the second person and introduced objects in relation to the listener’s suggested position in the environment. In addition, the allocentric description used canonical spatial terms (such as ‘north,’ ‘south,’ ‘east,’ and ‘west’) while the egocentric description used relative spatial terms (such as ‘to your left,’ ‘to your right,’ ‘in front of you,’ and ‘behind you’). Finally, the allocentric description first introduced the four major quadrants of the environment, then the individual objects (i.e., shops or animal cages) were mentioned: the organization was thus hierarchical. In contrast, the egocentric description started immediately with the first object and the overall layout of the environment was revealed in a step-wise, serial manner (i.e., linear organization).

Several factors, unrelated to reference frame, were held constant in all the descriptions. All objects were mentioned twice in each description. New locations were always introduced in reference to a previous location, ensuring that there were no discontinuities in the description. The number of words used in the English descriptions (for IW) was 318 in the allocentric description of the shopping center and 326 in the egocentric description of the zoo. The number of words used in the French descriptions was 360 in the allocentric description of the zoo, 310 in the egocentric description of the zoo, 292 in the allocentric description of the shopping center, and 378 in the egocentric description of the shopping center.

### Procedure

Each participant listened to two different descriptions out of four possible combinations (Allocentric-Shopping Center, Allocentric-Zoo, Egocentric-Shopping Center, or Egocentric-Zoo). Participants listened to the same description six times and were instructed to imagine and memorize the spatial environment and the location of the named objects. This experimental phase took approximately 15 min.

**FIGURE 2:**
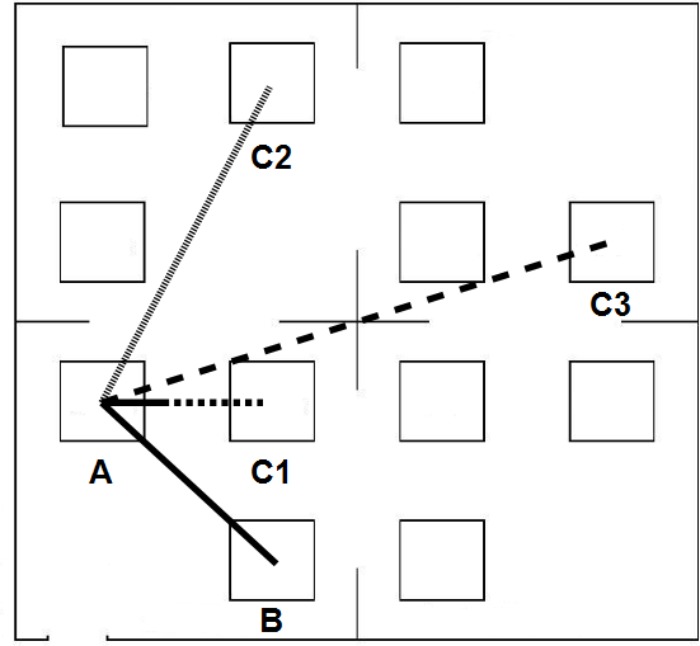
Participants had to compare two distances from a common starting location. In this example, the first distance is between objects A and B (AB). Consider that the second distance is between objects A and C3 (AC3): participants had to verbally report whether the distance AC3 was ‘longer’ or ‘shorter’ than the distance AB. Here, the distance AC3 is longer than the distance AB. Distance Differences were classified in three categories: Small, Medium, or Large. In this example, the distance difference between AC3 and AB is Large, the distance difference between AC2 and AB is Medium, and the distance difference between AC1 and AB is Small. A smaller distance difference is associated with a greater task difficulty, and thus with more errors.

Immediately after listening to the descriptions, the participants were asked to perform a distance-comparison task (Noordzij et al., 2006). To do so, participants were asked to picture a map of the environment and to mentally focus on the bird-flight distance that separated two enunciated objects. In addition, they were instructed that this distance would have to be compared to a second distance. Each trial started with a warning tone. Then two spoken names of objects were presented one after the other with a 300 ms gap in between. After a 2 s delay, participants heard another pair of objects’ names (with the same starting object as in the previous pair) and they had to answer whether the distance between the second pair of objects was longer or shorter than the first one by responding “longer” or “shorter,” respectively (**Figure 2**). The participants’ answers were recorded with the headset’s microphone. Participants had 12 s after the last object was named to give an answer, at which point the trial was terminated. The subsequent trial started when the participant was ready. Participants were first given two practice trials with feedback and then, as in Noordzij et al. (2006), they completed 48 experimental trials without feedback on their results. This experimental phase took approximately 20 min.

Two lists, one for the zoo, and one for the shopping center, were made of 48 pairs of two object’s names. These pairs had the first object’s name in common (e.g., “Chimpanzee-Hyena”/“Chimpanzee-Elephants”). Differences in distance between the two pairs were divided in three categories based on the difference on the printed map: Small difference (0–3 cm), Medium difference (3–6 cm), and Large difference (6–12 cm). There were 16 trials per Distance Difference. Trials were presented in a pseudorandom order, with the constraint that all three Distance Differences were presented in successive blocks of three trials. Each object was equally quoted during the task (for instance, “Chimpanzee” was used four times as the first common object name, four times as the second object, and four times as the last object).

The two deafferented participants first listened to the Allocentric-Shopping Center description, then to the Egocentric-Zoo description. For the sixteen control participants, the order of the allocentric and egocentric descriptions was counterbalanced (as were the shopping center and zoo descriptions) to assess potential effects of each type of description. This resulted in four groups of four healthy participants performing the task in one of the following condition: Allocentric-Shopping Center then Egocentric-Zoo, Allocentric-Zoo then Egocentric-Shopping Center, Egocentric-Shopping Center then Allocentric-Zoo, or Egocentric-Zoo then Allocentric-Shopping Center.

At the end of each distance-comparison task (i.e., with an Allocentric or Egocentric description), participants were asked to recall the objects on a printed 151 mm × 151 mm template of a map which presented all 12 locations, as in **Figure 2** except that no names appeared in the boxes. For the free-recall task, participants did not have any time constraint and were told to write in the correct location all the objects they could remember.

An additional test, the Minnesota Paper Form Board (Likert and Quasha, 1941), was used to assess visuo-spatial skills. This test, often used in imagery research (e.g., Denis and Cocude, 1997; Pazzaglia and De Beni, 2001), indicates with a score ranging from 0 to 31 (the greater, the better) the ability of an individual to mentally combine (using rotations) shapes in order to produce a reference shape. We were able to test the two deafferented participants as well as eleven controls out of the initial sixteen.

### Statistical Analyses

For all tests, the significance threshold was set at 0.05. For the free-recall task, *t*-tests for related samples were conducted on the control participants’ results to analyze the effect of the Reference Frame (Allocentric, Egocentric) on the number of objects placed in the correct location. For the distance-comparison task, repeated-measures analyses of variances (ANOVAs) were conducted to assess, for control participants, the influence of Reference Frame (Allocentric, Egocentric) and Distance Difference (Small, Medium, Large) on three dependant variables: error score (in percentage), reaction time (in seconds), and variability of reaction time (standard deviation of the mean, in seconds). For controls’ data, Statistica 8 (StatSoft, Tulsa, OK, United States) was used to perform ANOVAs. All data had normal distributions, as verified with the Kolmogorov–Smirnov method. Newman–Keuls tests were used for *post hoc* analysis.

Several statistical tests can be used to compare the performance of a single case to that of a group of controls. The *Q*′ test (Michael, 2007; Michael et al., 2009; Bartolo et al., 2018) was selected because, in addition to the case-group comparison, *Q*′ tests allow to test the significance of main and interaction effects in a 2×N, or even 2×2×N, statistical design. To do so, each deafferented participant’s mean value was transformed in a z score based on the mean and standard deviation of the values obtained with controls. The assessment of each case with respect to the controls was compared across experimental conditions. A 2×3 [Reference Frame (Allocentric, Egocentric) × 3 Distance Difference (Small, Medium, Large)] statistical design was used here.

## Results

### Free-Recall Task

#### Control Participants

**FIGURE 3:**
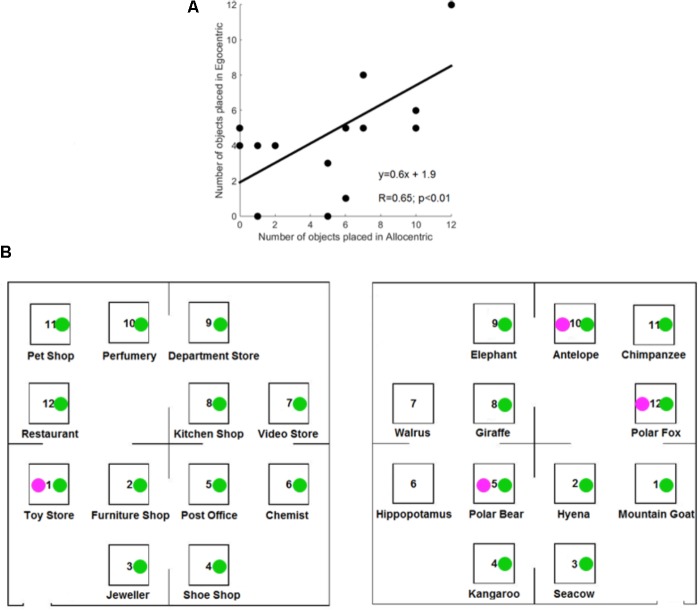
Results of the free-recall task in controls participants. **(A)** Correlation between the number of objects correctly placed in Allocentric and Egocentric conditions for the control participants. **(B)** Correctly located objects of GL (in magenta) and IW (in green).

Each of the result sections first focuses on data analyses for the group of control participants before addressing the influence of proprioceptive loss with deafferented individuals. On average, control participants correctly reported ∼5 objects (mean = 5.1) on a blank map after listening six times to a description. A *t*-test for related samples indicated that the number of objects placed in the correct location did not significantly differ between reference frames for control participants [*t*(15) = 0.6; *p* = 0.54]. **Figure 3A** illustrates the positive correlation between the number of objects placed in the correct location in the Allocentric and Egocentric conditions (*R* = 0.65; *p* < 0.01).

The number of correctly placed objects was subsequently analyzed to assess the possible influence of order. A 2×2 ANOVA [Reference Frame (Allocentric, Egocentric) × First Reference Frame Presented (Allocentric, Egocentric)] did not show any significant main effect nor interaction (each *p* > 0.1). A 2×2 ANOVA [Reference Frame (Allocentric, Egocentric) × First Environment Presented (Zoo, Shopping Center)] did not reveal any significant simple effect nor any interaction (each *p* > 0.2). The non-significant order effect allowed collapsing the data of all 16 participants into a single group, for all the subsequent analyses. An effect of serial position was found on the number of correctly located objects [*F*(11,341) = 3.6; *p* < 0.001]. *Post hoc* analysis revealed that the first, second, and final objects enumerated were better recalled by the participants than the seventh one. Also, the first, second, and final objects were better recalled than the tenth object. Overall, no factor (including age and gender) other than serial position appeared to significantly influence the number of correctly reported objects. In fact, neither age nor gender had a significant effect on any measure in the free-recall task or the distance-comparison task.

#### Deafferented Participants

**FIGURE 4:**
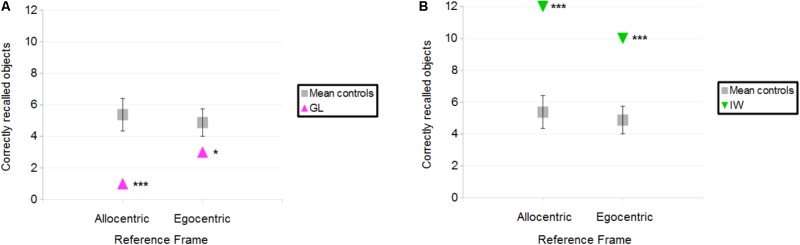
**(A)** Number of objects correctly placed for the control participants and deafferented participant GL as a function of Reference Frame. **(B)** Number of objects correctly placed for the control participants and deafferented participant IW. Error bars represent the standard error of the control group’s mean. ^∗^*p* < 0.05, ^∗∗∗^*p* < 0.001, significant difference.

**Figure 3B** illustrates the correctly located objects for the deafferented participants, and **Figure 4A** illustrates the finding that control participants reported more correctly located objects than the deafferented participant GL. Case-group comparisons showed the statistical significance of the findings in both the Allocentric condition (*Q*′ = −3.17; *p* < 0.001) and Egocentric condition (*Q*′ = −1.83; *p* < 0.05). The *Q*′ test showed no significant difference in the influence of Reference Frame between GL and the control group [*Q*′(1) = 1.78, *p* = 0.18; Cramer’s effect size: *V* = 0.33].

When considering IW, case-group comparisons showed that IW reported more correct objects than control participants in both Allocentric (*Q*′ = 3.97; *p* < 0.001) and Egocentric conditions (*Q*′ = 3.83; *p* < 0.001), as illustrated in **Figure 4B**. The *Q*′ test showed that when considering the influence of Reference Frame, IW’s pattern of results did not significantly differ from the controls’ pattern of results [*Q*′(1) = 0.64, *p* = 0.42; Cramer’s effect size: *V* = 0.2].

Case-group comparisons were used to assess whether GL or IW remembered as many objects as controls did (considering all remembered objects, even those not correctly located). There was no significant difference in remembered objects between GL (11) and controls (mean = 11.1) in the Allocentric condition (*Q*′ = ′0.35; *p* = 0.36), but GL remembered fewer objects (10) than controls (mean = 10.9) in the Egocentric condition (*Q*′ = −2.3; *p* < 0.05). A *Q*′ test showed no significant difference in the pattern of results between GL and controls [*Q*′(1) = 1.59; *p* = 0.2; Cramer’s effect size: *V* = 0.31]. IW remembered all 12 objects in both conditions and case-group comparisons showed that IW remembered more objects than controls in both Allocentric (*Q*′ = 2.29; *p* < 0.05) and Egocentric (*Q*′ = 2.55; *p* < 0.01) conditions. A *Q*′ test showed no significant difference between IW and controls’ patterns of results [*Q*′(1) = 0.08; *p* = 0.77; Cramer’s effect size: *V* = 0.07].

**Figure 3B** (right) illustrates that IW inverted two close objects in the Egocentric condition. To minimize the influence of such small errors on performance’s analysis, all participants’ data were also analyzed with the rule that an object was considered to be correctly located when it was in the correct corner of the fictitious map. Using this less stringent approach, the number of correctly located objects was still greater for controls in the Allocentric condition (mean = 7.9) compared to GL (number = 4; *Q*′ = −3.31; *p* < 0.001), but there was no significant difference in the Egocentric condition (controls’ mean = 6.8; vs. GL number = 6; *Q*′ = −0.84; *p* = 0.2). The number of correctly located objects was greater for IW compared to the controls in the Allocentric condition (number = 12; *Q*′ = 3.38; *p* < 0.001) and in the Egocentric condition (number = 12; *Q*′ = 4.04; *p* < 0.001).

#### Summary

In summary, the reference frame used for an environment description did not appear to influence performance in this free-recall spatial cognition task, as no significant differences were found between allocentric and egocentric conditions, neither for controls nor for deafferented participants. Controls were better than GL while IW was better than controls, suggesting that proprioception is not the sole factor influencing spatial cognition. While the results obtained in the free-recall task give an overall view of the quality of the spatial model derived from the auditory description, the distance-comparison task was expected to provide a more detailed analysis of the spatial model, and of the underlying mechanisms, for each individual.

### Distance-Comparison Task

#### Error Score

##### Control participants

**FIGURE 5:**
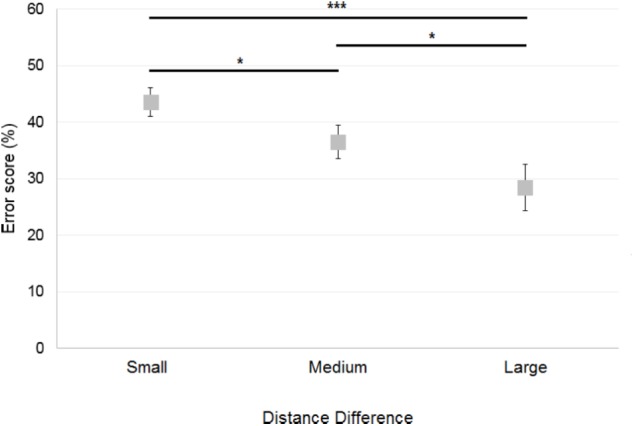
Mean error scores obtained by control participants across Distance Differences. Horizontal bars represent significant differences. Error bars represent standard errors. ^∗^*p* < 0.05, ^∗∗∗^*p* < 0.001, significant difference.

Trials in which the participants failed to provide a response before the trial was terminated were excluded from the analyses (note that there were only six such trials out of 1,728 in total). A 2×3 ANOVA [Reference Frame (Allocentric, Egocentric) × Distance Difference (Small, Medium, Large)] showed a significant main effect of Distance Difference [*F*(2,30) = 11; *p* < 0.001]. **Figure 5** illustrates this effect, which reflects the well-characterized finding that the smaller the difference between the two distances, the more difficult the comparison. The Distance Difference effect thus results in greater errors in the Small Distance Difference compared to the other conditions, as confirmed by *post hoc* analysis which showed that errors were greater when the Distance Difference was Small compared to when the Distance Difference was Medium (*p* < 0.05) and Large (*p* < 0.001). Errors were also greater when the Distance Difference was Medium compared to when the Distance Difference was Large (*p* < 0.05). There was no significant effect of Reference Frame [*F*(1,15) = 0.1; *p* = 0.74] and no significant interaction between Reference Frame and Distance Difference [*F*(2,30) = 0.47; *p* = 0.63].

Independent *t*-tests revealed no significant difference in error score as a function of the First Reference Frame Presented [*t*(14) = −0.05; *p* = 0.96] or First Description Presented [*t*(14) = −1.2; *p* = 0.24]. A *t*-test for related samples showed no significant effect of Environment [Zoo or Shopping Center; *t*(15) = −1.2; *p* = 0.24].

##### Deafferented participants

**FIGURE 6:**
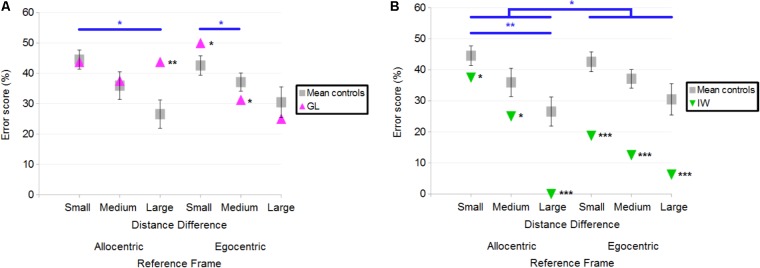
**(A)** Mean error scores for controls and deafferented participant GL across Distance Differences and Reference Frames. **(B)** Mean error scores for controls and deafferented participant IW across Distance Differences and Reference Frames. Error bars represent standard errors of the controls’ mean. Significant case-group comparisons are illustrated with black stars. Significant *Q***′** tests are illustrated with blue stars. ^∗^*p* < 0.05, ^∗∗^*p* < 0.01, ^∗∗∗^*p* < 0.001, significant difference.

The number of errors was used to compare the performance of each deafferented participant to that of age-matched controls. Case-group comparisons revealed that GL made significantly more errors than control participants in the Allocentric Reference Frame/Large Distance Difference (*Q*′ = 2.85; *p* < 0.01) and in the Egocentric/Small conditions (*Q*′ = 1.96; *p* < 0.05; **Figure 6A**). In the Egocentric/Medium condition, GL made less errors than controls (*Q*′ = −1.67; *p* < 0.05). The *Q*′ test showed that GL’s pattern of results did not significantly differ from that of the control group when considering the influence of Reference Frame [*Q*′(1) = 1.36; *p* = 0.24; Cramer’s effect size: *V* = 0.17] and Distance Difference [*Q*′(2) = 2.53; *p* = 0.28; Cramer’s effect size: *V* = 0.16]. Yet, the interaction Reference Frame × Distance Difference was significant [*Q*′(2) = 11.03; *p* < 0.01; Cramer’s effect size: *V* = 0.34], indicating that the pattern of results differed for controls and GL when considering Allocentric/Large and Allocentric/Small conditions (ψ = 2.61; *p* < 0.05). Indeed, **Figure 6A** illustrates that the difference between Allocentric/Small and Allocentric/Large conditions was greater for controls than for GL. Decomposition of the interaction also revealed a significant difference in the pattern of results between Egocentric/Small and Egocentric/Medium conditions (ψ = 2.78; *p* < 0.05), indicating that the change in performance was greater for GL compared to controls.

**Figure 6B** illustrates the fact that IW made fewer errors than control participants in all conditions. Case-group comparisons revealed that the differences were significant in the Allocentric/Small (*Q*′ = −1.87; *p* < 0.05), Allocentric/Medium (*Q*′ = −2.02; *p* < 0.05), Allocentric/Large (*Q*′ = −3.76; *p* < 0.001), Egocentric/Small (*Q*′ = −4.18; *p* < 0.001), Egocentric/Medium (*Q*′ = −4.28; *p* < 0.001) and Egocentric/Large (*Q*′ = −3.43; *p* < 0.001) conditions. The *Q*′ test revealed no significant effect of Distance Difference [*Q*′(2) = 2.71; *p* = 0.26; Cramer’s effect size: *V* = 0.17] but it showed an effect of Reference Frame [*Q*′(1) = 4.58; *p* < 0.05; Cramer’s effect size: *V* = 0.31]. **Figure 6B** illustrates this finding as the reduction of errors in Egocentric compared to Allocentric conditions was greater for IW compared to controls. The interaction Reference Frame × Distance Difference was significant [*Q*′(2) = 6.65; *p* < 0.01; Cramer’s effect size: *V* = 0.26], indicating that the change in errors for IW between Allocentric/Large and Allocentric/Small conditions was greater than that of controls (ψ = 3.45; *p* < 0.01).

#### Reaction Time

##### Control participants

**FIGURE 7:**
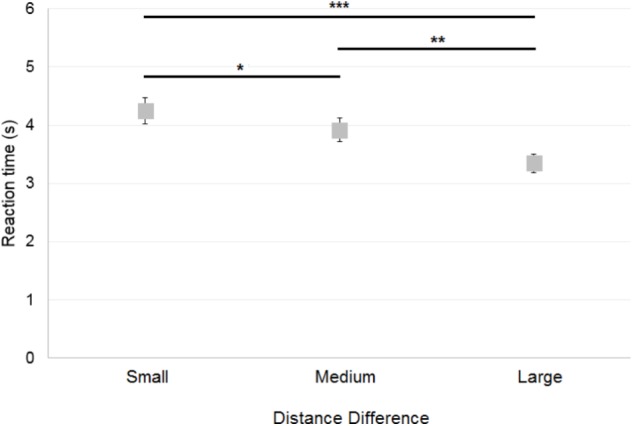
Mean reaction time of control participants across Distance Differences. Horizontal bars represent significant differences. Error bars represent standard errors. ^∗^*p* < 0.05, ^∗∗^*p* < 0.01, ^∗∗∗^*p* < 0.001, significant difference.

The reaction time across all correct trials (64% of trials) was analyzed. A 2×3 ANOVA [Reference Frame (Allocentric, Egocentric) × Distance Difference (Small, Medium, Large)] on the control participants’ reaction time showed a significant main effect of Distance Difference [*F*(2,30) = 18.2; *p* < 0.001; **Figure 7**], but no significant effect of Reference Frame [*F*(1,15) = 0.8; *p* = 0.38] and no significant interaction [*F*(2,30) = 0.6; *p* = 0.55]. *Post hoc* analysis showed that reaction time was greater when the Distance Difference was Small compared to Medium (*p* < 0.05) or Large (*p* < 0.001), as it can be seen on **Figure 7**. In addition, reaction time was greater when the Distance Difference was Medium compared to Large (*p* < 0.01).

No significant difference in reaction time was found as a function of First Reference Frame Presented [*t*(14) = −1.69; *p* = 0.11] and First Environment Presented [*t*(14) = −0.92; *p* = 0.37]. Also a *t*-test showed no significant effect of the Environment [Zoo or Shopping Center; *t*(15) = −1.43; *p* = 0.17]. Similar findings were found when correct and incorrect responses were pooled. Note that a *t*-test for related samples showed that the reaction time of incorrect responses (mean = 4.185 ± 0.938 s) was greater than that of correct responses [mean = 3.736 ± 0.749 s; *t*(15) = −2.58; *p* < 0.05].

##### Deafferented participants

**FIGURE 8:**
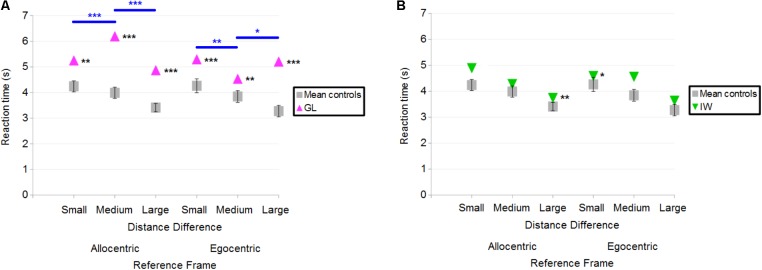
**(A)** Mean reaction time of control participants and deafferented participant GL across Distance Differences and Reference Frames. **(B)** Mean reaction time of control participants and deafferented participant IW across Distance Differences and Reference Frames. Error bars represent standard errors of the controls’ mean. Significant case-group comparisons illustrated with black stars. Significant *Q*′ tests illustrated with blue stars. ^∗^*p* < 0.05, ^∗∗^*p* < 0.01, ^∗∗∗^*p* < 0.001, significant difference.

Overall, both GL and IW had longer reaction times (in correct trials) than control participants. **Figure 8A** illustrates the case-group comparisons and the statistically significant finding that GL was slower than control participants in all conditions [Allocentric Reference Frame/Small Distance Difference (*Q*′ = 3.01; *p* < 0.01), Allocentric/Medium (*Q*′ = 4.43; *p* < 0.001), Allocentric/Large (*Q*′ = 4.06; *p* < 0.001), Egocentric/Small (*Q*′ = 4.3; *p* < 0.001), Egocentric/Medium (*Q*′ = 2.6; *p* < 0.01) and Egocentric/Large (*Q*′ = 4.24; *p* < 0.001)]. The *Q*′ test showed no significant effect of the Reference Frame [*Q*′(1) = 1; *p* = 0.32; Cramer’s effect size: *V* = 0.15] and Distance Difference [*Q*′(2) = 1.89; *p* = 0.39; Cramer’s effect size: *V* = 0.14]. However, the interaction Reference Frame × Distance Difference was significant [*Q*′(2) = 47.19; *p* < 0.001; Cramer’s effect size: *V* = 0.72]. This indicated that the pattern of results for GL differed from that of controls between Allocentric/Small and Allocentric/Medium conditions (ψ = 4.43; *p* < 0.001): **Figure 8A** shows that the reaction time decreased for controls but not for GL. In addition, the change in reaction time between Allocentric/Medium and Allocentric/Large conditions (ψ = 6.34; *p* < 0.001) was greater for GL than for controls. Moreover, the pattern of results for GL differed from that of controls between Egocentric/Small and Egocentric/Medium conditions (ψ = 3.66; *p* < 0.01) as the change in reaction time was greater for GL than for controls. The pattern of results for GL also differed from that of controls between Egocentric/Medium and Egocentric/Large conditions (ψ = 2.67; *p* < 0.05) as reaction time decreased for controls but not for GL.

**Figure 8B** illustrates the case-group comparisons and the finding that IW was significantly slower than control participants in the Allocentric/Large (*Q*′ = 2.59; *p* < 0.01) and in the Egocentric/Small (*Q*′ = 1.87; *p* < 0.05) conditions. The *Q*′ test showed no significant effect of Reference Frame [*Q*′(1) = 0.75; *p* = 0.39; Cramer’s effect size: *V* = 0.12], Distance Difference [*Q*′(2) = 0.57; *p* = 0.75; Cramer’s effect size: *V* = 0.08] and no significant interaction [*Q*′(2) = 2.75; *p* = 0.25; Cramer’s effect size: *V* = 0.17]. Similar findings were observed for the global reaction time including correct and incorrect responses.

Overall, IW gave more correct responses but was slower to answer compared to controls in the distance-comparison task. To take into account the speed-accuracy trade-off, a composite measure was used, which consisted in the number of correct responses divided by the mean reaction time. This was separately calculated for each experimental condition, for IW and the controls. Using this ratio, case-group comparisons revealed that IW performance remained significantly better than controls in Allocentric/Small (mean = 2.4 correct responses/s; controls’ mean = 2.1; *Q*′ = 2.00; *p* < 0.05) and Egocentric/Small (mean = 2.8 correct responses/s; controls’ mean = 2.2; *Q*′ = 1.94; *p* < 0.05) conditions. IW performance was marginally better in Allocentric/Large (mean = 4.3 correct responses/s; controls’ mean = 3.6; *Q*′ = 1.64; *p* = 0.051) and Egocentric/Medium (mean = 3.4 correct responses/s; controls’ mean = 2.7; *Q*′ = 1.38; *p* = 0.083) conditions. The *Q*′ test showed no significant effect of the Reference Frame [*Q*′(1) = 0.14; *p* = 0.71; Cramer’s effect size: *V* = 0.05], Distance Difference [*Q*′(2) = 0.36; *p* = 0.36; Cramer’s effect size: *V* = 0.06] and no significant interaction [*Q*′(2) = 0.53; *p* = 0.53; Cramer’s effect size: *V* = 0.08]. Note that normalizing GL’s reaction times by performance would only increase the difference from the controls.

#### Variability of Reaction Time

##### Control participants

**FIGURE 9:**
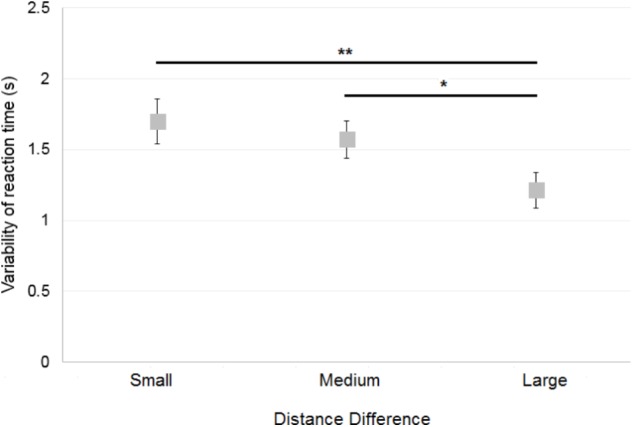
Mean variability of the control participants’ reaction time across Distance Differences. Horizontal bars represent significant differences. Error bars represent standard errors. ^∗^*p* < 0.05, ^∗∗^*p* < 0.01, significant difference.

Variability of reaction time was assessed by computing the standard deviation of the mean reaction time of correct responses. When first considering data of the control participants only, a 2×3 ANOVA [Reference Frame (Allocentric, Egocentric) × Distance Difference (Small, Medium, Large)] showed a significant main effect of Distance Difference [*F*(2,30) = 5.9; *p* < 0.01; **Figure 9**], but no significant effect of Reference Frame [*F*(1,15) = 0.5; *p* = 0.5] and no significant interaction [*F*(2,30) = 0.2; *p* = 0.79]. *Post hoc* analysis showed that variability of reaction time was greater when the Distance Difference was Small compared to Large (*p* < 0.01) and that variability of reaction time was greater when the Distance Difference was Medium compared to Large (*p* < 0.05). No significant effect was found for the Environment [Zoo or Shopping Center; *t*(15) = −1.09; *p* = 0.29], First Environment Presented [*t*(14) = 0.06; *p* = 0.95] and for First Reference Frame Presented [*t*(14) = −1.58; *p* = 0.14].

##### Deafferented participants

**FIGURE 10:**
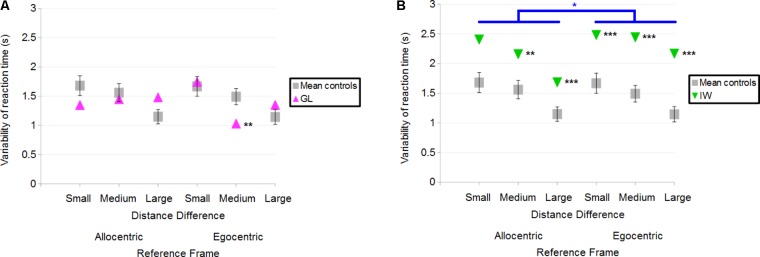
**(A)** Mean variability of reaction time for the control participants and deafferented participant GL across Distance Differences and Reference Frames. **(B)** Mean variability of reaction time for the control participants and deafferented participant IW across Distance Differences and Reference Frames. Error bars represent standard errors of the controls’ mean. Significant case-group comparisons are illustrated with black stars. Significant *Q*′ tests are illustrated with blue stars. ^∗^*p* < 0.05, ^∗∗^*p* < 0.01, ^∗∗∗^*p* < 0.001, significant difference.

Overall, GL had a variability of reaction time similar to controls. When considering each specific condition in case-group comparisons, results (illustrated in **Figure 10A**) showed that only one significant difference could be found between GL and controls: GL had a smaller variability of reaction time than control participants in the Egocentric/Medium condition (*Q*′ = −2.57; *p* < 0.01). The *Q*′ test showed no significant effect of the Reference Frame [*Q*′(1) = 0.02; *p* = 0.89; Cramer’s effect size: *V* = 0.02], Distance Difference [*Q*′(2) = 3.15; *p* = 0.21; Cramer’s effect size: *V* = 0.18] and no significant interaction [*Q*′(2) = 5.35; *p* = 0.07; Cramer’s effect size: *V* = 0.23].

Case-group comparisons showed that IW’s reaction times were more variable than that of controls, as illustrated in **Figure 10B** [Allocentric Reference Frame/Small Distance Difference (*Q*′ = 1.52; *p* = 0.06), Allocentric/Medium (*Q*′ = 2.82; *p* < 0.01), Allocentric/Large (*Q*′ = 3.45; *p* < 0.001), Egocentric/Small (*Q*′ = 3.77; *p* < 0.001), Egocentric/Medium (*Q*′ = 3.38; *p* < 0.001) and the Egocentric/Large condition (*Q*′ = 4.25; *p* < 0.001)]. The *Q*′ test showed a significant effect of Reference Frame [*Q*′(1) = 6.54; *p* < 0.05; Cramer’s effect size: *V* = 0.37] indicating that IW’s variability of reaction time changed more between Egocentric and Allocentric conditions compared to controls. No significant effect of Distance Difference was found [*Q*′(2) = 0.12; *p* = 0.94; Cramer’s effect size: *V* = 0.03] and no significant interaction either [*Q*′(2) = 3.21; *p* = 0.2; Cramer’s effect size: *V* = 0.18].

#### Summary

In the distance comparison task, control participants made less errors as the Distance Difference increased. Similarly, an increase in Distance Difference led to a decrease in the reaction time and in the variability of reaction time. The Reference Frame did not significantly influence any of the dependent variables in this task. It is, however, difficult to conclude on the influence of proprioceptive loss on the quality of the spatial representation, as the two individuals differed from the controls in opposite directions: indeed, GL made more errors, especially in the Allocentric/Large condition, and IW made less errors than controls in all conditions. However, we found that for both deafferented participants, reaction time was altered compared to controls, even when controlling for any potential speed-accuracy trade-off.

### Minnesota Paper Form Board (MPFB)

#### Control Participants

**FIGURE 11:**
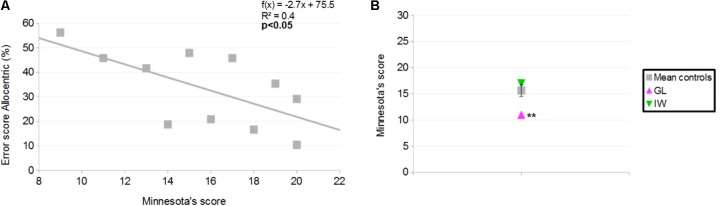
**(A)** Correlation between Minnesota Paper Form Board (MPFB)’s score (x) and error score in the Allocentric condition of the distance-comparison task, for controls (y). **(B)** MPFB’s score for the controls and the deafferented participants. Error bars represent standard errors of controls’ mean. ^∗∗^*p* < 0.01, significant difference.

Mean score for controls was 15.8 ± 3.8 correct responses (31 was the best possible score and scores ranged between 9 and 20). A significant, negative correlation was found between the MPFB’s score and the error score in the Allocentric condition for the distance-comparison task (*r* = −0.6; *p* < 0.05; **Figure 11A**). This indicates that for controls, the greater the performance in the MPFB test, the greater the performance in the distance-comparison task in the Allocentric condition.

#### Deafferented Participants

GL scored 11 in the Minnesota Paper Board Form and IW scored 17. Case-group comparisons showed that GL’s score was smaller than the mean score of controls (*Q*′ = −2.95; *p* < 0.01; **Figure 11B**) and that no significant difference was found between IW’s score and the mean score of controls (*Q*′ = 0.92; *p* = 0.18).

### Neuropsychological Assessment of GL Memory and Attention

GL’s performance in the free-recall task was impaired with respect to controls, while IW outperformed controls. In the distance-comparison task, GL made more errors than controls in a couple of experimental conditions. To explore the possibility that GL’s impairments were related to memory impairments, a neuropsychologist assessed GL’s memory with the Wechsler Memory Scale-III (WMS-III; Wechsler, 2001). GL received an overall score of 106 for her general memory capacities, and a score of 105 for her working memory. With respect to normal data of age-matched controls (score = 100 ± 15), this corresponds to the 66th and 63rd percentile, respectively. No significant impairment was detected in subtests of logical memory (immediate auditory recall = 10; delayed auditory recall = 11; general memory recall = 11) compared to controls (score = 10 ± 3). To explore the possibility that GL’s impairments were related to attention impairments, GL’s sustained attention was assessed with a Test Battery of Attentional Performance (TAP version 2.2; Zimmermann and Fimm, 2002). GL’s total score corresponded to the 54th percentile, i.e., attention capacities were similar to age-matched controls. Overall, the neuropsychological assessment of GL revealed normal memory and attention capacities, suggesting that memory and attention issues are unlikely to explain GL’s impairments in the spatial cognition tasks.

## Discussion

This study investigated how proprioception might influence the ability to form spatial mental models and the ability to infer spatial relationships from these. Using methods similar to Noordzij et al. (2006), performance of two chronically deafferented participants and of a control group were compared in a distance-comparison task and a free-recall task after they listened to an auditory description of a spatial environment. The type of description was presented according to either an egocentric or an allocentric reference frame. Participants were also assessed on a visuo-spatial test: the Minnesota Paper Form Board.

The distance-comparison task was difficult, as reflected by the high global error score of control participants (36.2%), which was similar to that obtained in the Noordzij and Postma (2005) and Noordzij et al. (2006) studies. Elderly adults have been found to be impaired compared to young adults when they have to form a mental model from a description that contains four elements (Copeland and Radvansky, 2007), so substantial errors were expected in our 12 elements description. When control participants had to compare two distances, a main effect of the distance difference was observed: as in previous work, the larger the distance difference, the smaller the error score. This effect has been described as the “symbolic distance effect” (Denis and Zimmer, 1992; Afonso et al., 2003; Denis, 2008) and our work confirms that even without metrical information in the description of a spatial environment, participants can infer distance information from a recently formed spatial model. Results from the distance-comparison task and from the free-recall task did not reveal any significant difference between allocentric and egocentric conditions, consistent with previous work (Tversky, 1991; Taylor and Tversky, 1992). This suggests that more work is necessary to determine the influence of a description’s reference frame on the formation of a spatial mental model. A different task may be tested as, in the present study and in previous work (Noordzij and Postma, 2005; Noordzij et al., 2006), participants were asked in the distance-comparison task to focus on the bird-flight distance that separated two enunciated objects. Such instruction may have resulted in a recoding of the spatial representation built during the egocentric condition into an allocentric reference frame. Such recoding may explain the positive correlation between recalled objects in allocentric and egocentric reference frames and the non-significant effect of reference frame in the distance-comparison and free-recall tasks.

Since visual loss has been shown to influence the formation of spatial models (see for instance Noordzij et al., 2006; Pasqualotto et al., 2013), we hypothesized that proprioceptive loss might also influence the formation of spatial models. Such sensory impairment could interact with the used reference frames given the various specificities of sensory systems. Indeed, it has been suggested that visual information is initially coded in retinotopic coordinates, auditory information is initially coded in head-centered coordinates, whereas proprioceptive and tactile information are initially coded in somatotopic coordinates (Cohen and Andersen, 2002, see also O’Brien and Auvray, 2016, for a discussion). Studying this issue is complex because several other factors interact. For instance, the spatial coordinates of a tactile stimulus can be referenced to the stimulated body part, to the entire body, or to the external world (Arnold et al., 2016, 2017b; Arnold and Auvray, 2017) and these different reference frames can conflict, for instance when presenting tactile stimuli to crossed arms (Shore et al., 2002). However challenging in what follows we discuss the possible influences of several inter-individual differences (and in particular the differences between the two deafferented participants) in addition to the effect of the loss of proprioception.

The first main result to emerge from our study is that despite a massive loss of proprioception, deafferented individuals can form a reasonably good mental model based on a verbal description formatted in either allocentric or egocentric reference frames. In most experimental conditions, the two deafferented participants made less than 50% of errors (chance level when considering that participants had to respond either ‘Longer’ or ‘Shorter’). Such performance was surprising in particular in the egocentric condition because previous work on GL highlighted how the loss of proprioception could impair the representation of self attributes (Blouin et al., 1993; Ghez et al., 1995). In the present study, GL generally performed worse than controls. After the experiment, GL acknowledged struggling with the terms “east” and “west,” suggesting some degree of uncertainty in the allocentric representation. This may be linked to the observation that in contrast to the egocentric condition, her error scores across the three distance differences in the allocentric condition did not show the expected linear trend, which may suggest an impaired formation of the spatial model in an allocentric reference frame. This appears to be supported by the small number of objects GL correctly reported in the free-recall task. GL’s deficit in the allocentric condition may be linked to difficulties in visuo-spatial imagery, which were evidenced with the Minnesota Paper Form Board: in this visuo-spatial test, her score was significantly lower than that of controls.

We had the rare opportunity to work with another, well-characterized deafferented individual. IW performed better than controls in the free-recall task but also in the distance-comparison task when considering the error score. Thus, a massively deafferented individual can build an accurate spatial model. The fact that IW could outperform healthy controls in a spatial cognition task may be related to his ‘cognitive style of life’ and also to his usually cautious approach. Since he lost proprioception, IW has been using huge mental efforts to perform daily activities and in particular to control his movements (Cole, 1995, 2016). Such reliance on cognitive resources was highlighted by Ingram et al. (2000) who studied IW’s motor performance in conditions with and without a concurrent, arithmetic task. Their results showed that IW’s motor performance was severely impaired when he had to divide his attention with a counting backward task.

Cole et al. (1995) highlighted the fact that IW could focus his attention more successfully than normal controls could. This capacity to focus attention may be linked to an increased working memory, which could influence visuo-spatial abilities (Farmer et al., 1986; Garden et al., 2002). Working memory is an individual feature that has been linked to visuo-spatial abilities as, for instance, dual-task studies reported that performance in visuo-spatial tasks is impaired when working memory is loaded with secondary verbal or spatial tasks (Farmer et al., 1986; Garden et al., 2002; Meilinger et al., 2008). Also, working memory has been found to be positively correlated to visuo-spatial abilities across individuals (Hegarty et al., 2006). Here we did not find a working memory deficit for GL and therefore cannot link her working memory capacities with her results in the distance comparison task. One should address the hypothesis that IW has a better than average working memory capacity, which may explain the accuracy of his spatial representations. Further tests are thus needed on IW and healthy controls to precisely characterize the influence of working memory on spatial cognition.

IW, after his proprioceptive loss, has been able to drive again and he developed a company whose aim is to assess how accessible public and private buildings are for the disabled. IW has used GPS and maps, and he likely developed his visuospatial skills, as highlighted by the fact that he has vivid memories of places such as hotels or movie theaters that he visited many years previously (Cole, 2016). In addition, ter Horst et al. (2012) showed that IW’s visual imagery processes were enhanced compared to controls. It remains unclear which specific individual difference is key when comparing IW and GL but here, differences in task performance may be explained at least partly by visuo-spatial imagery as GL was a low performer, not IW, in the Minnesota Paper Form Board Test. Denis (2008) previously reported that individuals who performed well in such a visuo-spatial test also performed well in a distance-comparison task. In line with this finding, we found a positive correlation between the Minnesota Paper Form Board’s score and the error score in the Allocentric condition of the distance-comparison task. Neck proprioception (present for IW but absent for GL) may also partly explain the differences found in the present study. In the future, it will be interesting to assess more thoroughly the attentional and visuospatial skills of these, and additional, deafferented individuals compared to controls.

One factor that should be taken into account, and further studied, is the influence of being a wheelchair user on spatial cognition. Both GL and IW have been using the wheelchair for years: GL ever since her proprioceptive loss while IW used a wheelchair for the first few years, then abandoned it as he regained the ability to walk. IW eventually returned to wheelchair use about 17 years ago to ease back pain and has used it full time for the last 10 years. It is possible that using a wheelchair influences the preferred reference frame, and thus spatial cognition. In fact, and of interest for the present study, there is evidence that spinal cord injury, which often leads individuals to use a wheelchair, can result in cognitive impairments such as deficits in visuospatial perception (Davidoff et al., 1992; Craig et al., 2017).

The second main result to emerge from our study is that both deafferented participants had a higher overall reaction time than controls. This is in line with findings of Lajoie et al. (1996), who reported that IW responded later to a tone while walking compared to controls. This result suggested that he uses a substantial amount of attention to control posture and gait, something that IW acknowledges. Even sitting in a wheelchair requires attention for IW and GL to maintain posture, and the only time when such proprioceptively deafferented individuals are freed of this kind of attention requirements is when lying in a secure bed (Cole, 2016). Since both deafferented individuals performed the task while sitting, perhaps this postural control represented a dual task, hence their higher reaction time compared to controls. Alternatively, the loss of proprioception could specifically impact the reaction time when a participant has to infer spatial relationships from a spatial model. The loss of proprioception has been shown to impact the timing in sensorimotor tasks (Bard et al., 1992; Sainburg et al., 1993), and further experiments should be conducted to assess whether such loss directly influences spatial cognition. Here, GL’s longer reaction times may be due to the difficulty of the cognitive task, as she made more errors than controls. On the other hand, IW made less errors than controls. One possibility is that his longer reaction time reflects a conservative strategy to perform the task, something that IW confirmed afterward, acknowledging that he developed a natural tendency to take his time to perform well. The analysis of reaction time variability offered some insights on this possible strategy. Reaction time was found to be more variable for IW compared to controls in all experimental conditions, and further analysis revealed that IW appeared to respond quickly (approximately in 2 s) when, presumably, he was certain about his answer (although it could be correct or incorrect) while when he was uncertain, he appeared to take more time than controls before finally responding. This likely explains the increase in his mean and variable reaction times.

In summary and to respond to the question ‘Does proprioception influence spatial cognition?’ our findings on reaction time indicate that proprioception can have an impact on performance in a spatial cognition task, extending previous work by ter Horst et al. (2012). However, despite this increase in reaction times for both deafferented participants, the quality of their spatial representation differed, indicating that other factors such as attention, memory, and visuo-spatial abilities may all contribute to spatial cognitive skills.

## Author Contributions

AR, MA, and FS designed the experiments. AR and GP coded for the experiments. AR performed the experiments and prepared the figures. AR and FS analyzed the data and drafted the manuscript. AR, MA, GP, RM, JC, and FS interpreted results of experiments, edited the manuscript, and approved the final version for submission.

## Conflict of Interest Statement

The authors declare that the research was conducted in the absence of any commercial or financial relationships that could be construed as a potential conflict of interest.

## APPENDIX

### Description of the Shopping Center in an Allocentric Reference Frame

The shopping center is a square and is divided into four zones. The first zone is the southwest corner of the shopping center, the second zone is the southeast corner of the shopping center, the third zone is the northeast corner of the shopping center, and the fourth zone is the northwest corner of the shopping center. There are three stores in each zone and these stores are all squares of the same size. The entrance is on the west side of the south wall and the entrance is pointed to the north.

The **toy store** is in the northwest corner of the first zone. To the east of the **toy store** is the **furniture shop**, in the northeast corner of the first zone. To the south of the **furniture shop** is the **jeweler**, in the southeast corner of the first zone.

To the east of the **jeweler** is the **shoe shop**, in the southwest corner of the second zone. To the north of the **shoe shop** is the **post office**, in the northwest corner of the second zone. To the east of the **post office** is the **chemist**, in the northeast corner of the second zone.

To the north of the **chemist** is the **video store**, in the southeast corner of the third zone. To the west of the **video store** is the **kitchen shop**, in the southwest corner of the third zone. To the north of the **kitchen shop** is the **department store**, in the northwest corner of the third zone.

To the west of the **department store** is the **perfumery**, in the northeast corner of the fourth zone. To the west of the **perfumery** is the **pet shop**, in the northwest corner of the fourth zone. To the south of the **pet shop** is the **restaurant**, in the southwest corner of the fourth zone. To the south of the **restaurant** is the first zone again.

### Description of the Shopping Center in an Egocentric Reference Frame

In the shopping center, there are stores. These are all isolated square units of the same size. You enter the shopping center in the first zone and in front of you is the **toy store**. You walk toward the **toy store** and in front of the **toy store** you turn to the right with an angle of 90 degrees and then you walk straight on. Next, you come to the **furniture shop** on your left and the **jeweler** on your right.

You walk straight in between the **furniture shop** and the **jeweler** and then you come to the **postal office** on your left and the **shoe shop** on your right. You are now in the second zone of the shopping center. You walk straight with the **shoe shop** still on your right and then you turn left with an angle of 90 degrees. The **postal office** is still on your left and the **chemist** is to your right.

You walk straight with the **chemist** on your right and then you come to the **video store** on your right and the **kitchen shop** on your left. You are now in the third zone of the shopping center. You walk straight with the **video store** on your right and then you turn left with an angle of 90 degrees. The **kitchen shop** is still on your left and the **department store** is now on your right.

You walk straight with the **department store** on your right and then you come to the **perfumery** on your right. You are now in the fourth zone of the shopping center. You walk straight with the **perfumery** on your right and in front of you to the right is the **pet shop**. You then turn left with an angle of 90 degrees such that the **pet shop** is behind you to the right and the **restaurant** is now directly to your right. You walk straight with the **restaurant** on your right. If you keep walking straight, you are back in the first zone.

### Description of the Zoo in an Allocentric Reference Frame

The zoo is a square and is divided into four zones. The first zone is the southeast corner of the zoo, the second zone is the southwest corner of the zoo, the third zone is the northwest corner of the zoo, and the fourth zone is the northeast corner of the zoo. There are three cages with animals in each zone, and these cages are all squares of the same size. The entrance is on the east side of the south wall and the entrance is pointed to the north.

The **mountain goats** are in the northeast corner of the first zone. To the west of the **mountain goats** are the **hyenas**, in the northwest corner of the first zone. To the south of the **hyenas** are the **sea cows**, in the southwest corner of the first zone.

To the west of the **sea cows** are the **kangaroos**, in the southeast corner of the second zone. To the north of the **kangaroos** are the **polar bears**, in the northeast corner of the second zone. To the west of the **polar bears** are the **hippopotamus**, in the northwest corner of the second zone.

To the north of the **hippopotamus** are the **walrus**, in the southwest corner of the third zone. To the east of the **walrus** are the **giraffes**, in the southeast corner of the third zone. To the north of the **giraffes** are the **elephants**, in the northeast corner of the third zone.

To the east of the **elephants** are the **antelopes**, in the northwest corner of the fourth zone. To the east of the **antelopes** are the **chimpanzees**, in the northeast corner of the fourth zone. To the south of the **chimpanzees** are the **polar foxes**, in the southeast corner of the fourth zone. To the south of the **polar foxes** is the first zone again.

### Description of the Zoo in an Egocentric Reference Frame

In the zoo, there are enclosed cages. These are all isolated square units of the same size. You enter the zoo in the first zone and in front of you are the **mountain goats**. You walk toward the **mountain goats** and in front of the **mountain goats** you turn to your left with an angle of 90 degrees and then you walk straight on. Next, you come to the **sea cows** on your left and the **hyenas** on your right.

You walk straight in between the **sea cows** and the **hyenas** and then you come to the **kangaroos** on your left and the **polar bears** on your right. You are now in the second zone of the zoo. You walk straight with the **kangaroos** still on your left and then you turn right with an angle of 90 degrees. The **polar bears** are still on your right and the **hippopotamuses** are now on your left.

You walk straight with the **hippopotamuses** on your left and then you come to the **walruses** on your left and the **giraffes** on your right. You are now in the third zone of the zoo. You walk straight with the **walruses** on your left and then you turn right with an angle of 90 degrees. The **giraffes** are still on your right and the **elephants** are now on your left.

You walk straight with the **elephants** on your left and then you come to the **antelopes** on your left. You are now in the fourth zone of the zoo. You walk straight with the **antelopes** on your left and in front of you to the left are the **chimpanzees**. You then turn right with an angle of 90 degrees such that the **chimpanzees** are behind you to the left and the **polar foxes** are now directly to your left. You walk straight with the **polar foxes** on your left. If you keep walking straight, you are back in the first zone.
